# Application of Deep Learning for Prediction of Alzheimer’s Disease in PET/MR Imaging

**DOI:** 10.3390/bioengineering10101120

**Published:** 2023-09-24

**Authors:** Yan Zhao, Qianrui Guo, Yukun Zhang, Jia Zheng, Yang Yang, Xuemei Du, Hongbo Feng, Shuo Zhang

**Affiliations:** 1 Department of Information Center, The First Affiliated Hospital, Dalian Medical University, Dalian 116011, China; 2 Department of Nuclear Medicine, Beijing Cancer Hospital, Beijing 100142, China; guoqianrui0154@163.com; 3 Department of Radiology, The First Affiliated Hospital, Dalian Medical University, Dalian 116011, China; 4 Department of Nuclear Medicine, The First Affiliated Hospital, Dalian Medical University, Dalian 116011, China; 5 Beijing United Imaging Research Institute of Intelligent Imaging, Beijing 100094, China

**Keywords:** Alzheimer’s disease, deep learning, positron emission tomography, magnetic resonance

## Abstract

Alzheimer’s disease (AD) is a progressive neurodegenerative disorder that affects millions of people worldwide. Positron emission tomography/magnetic resonance (PET/MR) imaging is a promising technique that combines the advantages of PET and MR to provide both functional and structural information of the brain. Deep learning (DL) is a subfield of machine learning (ML) and artificial intelligence (AI) that focuses on developing algorithms and models inspired by the structure and function of the human brain’s neural networks. DL has been applied to various aspects of PET/MR imaging in AD, such as image segmentation, image reconstruction, diagnosis and prediction, and visualization of pathological features. In this review, we introduce the basic concepts and types of DL algorithms, such as feed forward neural networks, convolutional neural networks, recurrent neural networks, and autoencoders. We then summarize the current applications and challenges of DL in PET/MR imaging in AD, and discuss the future directions and opportunities for automated diagnosis, predictions of models, and personalized medicine. We conclude that DL has great potential to improve the quality and efficiency of PET/MR imaging in AD, and to provide new insights into the pathophysiology and treatment of this devastating disease.

## 1. Introduction

Alzheimer’s disease (AD) is a progressive neurodegenerative disorder that detrimentally impacts cognitive function, memory, and behavior. This devastating condition is primarily characterized by the accumulation of amyloid-beta (Aβ) plaques and tau protein tangles within the brain [1,2]. It stands as the predominant cause of dementia, responsible for 60–80% of all cases [3]. Alarmingly, its prevalence is projected to triple globally by 2050, with AD estimates reaching threefold higher rates [4]. Given the absence of a cure and the limited efficacy of available treatments in halting symptom progression, early detection and intervention play a pivotal role in managing AD [5].

In the study of AD and mild cognitive impairment (MCI), non-invasive imaging techniques such as positron emission tomography (PET) and magnetic resonance imaging (MRI) have been instrumental in unraveling insights into brain structure and function [6,7,8]. PET imaging, for instance, facilitates the identification of Aβ protein accumulation [9,10], while MRI provides high-resolution structural brain images capable of detecting AD-related atrophy in specific brain regions [11]. Nonetheless, the analysis of PET and MRI images in AD and MCI poses challenges due to subtle cerebral changes and discrepancies among observers. Consequently, there exists a pressing need for objective and quantitative techniques that can enhance the early detection of AD and MCI.

**Figure 1:**
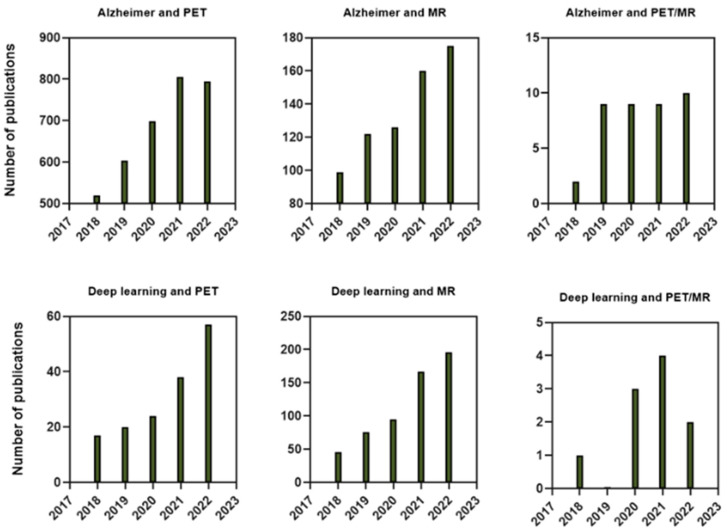
The number of publications in the Web of Science related to Alzheimer’s disease, deep learning, and PET/MR imaging were analyzed by year.

Recent publications [12,13,14] have witnessed a burgeoning interest in the utilization of deep learning (DL) algorithms for the analysis of medical images. The number of publications in the Web of Science related to Alzheimer’s disease, deep learning, and PET/MR imaging, as depicted in Figure 1. DL offers the advantage of automatically learning features from extensive datasets, obviating the need for manual feature extraction [15,16,17,18]. This not only reduces the time and effort required for image analysis but also improves accuracy [19,20,21]. Moreover, DL algorithms can be employed for image segmentation, a critical task in identifying specific regions of interest during medical imaging analysis. Nevertheless, further research is imperative to validate the efficacy of these algorithms in real-world clinical settings.

In this paper, we introduce several innovative characteristics in the application of DL for the prediction of AD in PET/MR imaging. We introduce novel DL models that leverage the power of artificial intelligence to analyze complex imaging data and accurately predict the presence and progression of AD. Additionally, we highlight the importance of multimodal imaging, combining PET and MR scans, to enhance the accuracy and reliability of the predictions. We also discuss the potential of automated diagnosis and personalized medicine in optimizing the performance of DL models for Alzheimer’s disease prediction. Overall, we present cutting-edge advancements and novel approaches that contribute to the field of DL in AD research.

The paper begins by discussing the significance of PET/MR imaging in AD research. It then introduces various DL algorithms. The paper further explores the application of DL in AD PET/MR imaging, focusing on image segmentation, image reconstruction, diagnosis and prediction, and visualization of pathological features. Finally, the paper suggests future directions for research in this area.

## 2. PET/MR Imaging in AD

The early detection of AD is of paramount importance for effective patient management and prognostication. PET tracers for glucose metabolism [8,22,23,24], amyloid [25,26], tau [27,28,29,30,31,32], and neuroinflammation imaging [33,34,35,36,37,38,39], as well as MRI techniques such as arterial spin labeling (ASL) [40,41,42], resting-state fMRI and task-related fMRI [43,44,45,46,47,48,49,50,51,52], multi-nuclear MRI [53,54], and chemical exchange saturation transfer (CEST) [55,56], have provided valuable insights into the pathological mechanisms of AD in patients (Table 1).

Synapse loss is a major pathological change in AD, but its relationship to functional and structural connectivity dysfunction remains unclear. In the latest published research [57], 18F-SynVesT-1 PET/MR was used to measure synaptic vesicle glycoprotein 2 A (SV2A) binding and evaluate synaptic alterations in participants with AD, MCI, and controls. The PET and MRI data were acquired simultaneously on the United Imaging uPMR790 system. Compared to controls, lower synaptic density was found in the cortex and hippocampus of the AD group. Cognitive decline was correlated with synaptic density changes in the right insular cortex and bilateral caudal middle frontal gyrus (MFG). Specifically, the synaptic density in the right MFG was positively associated with functional connectivity between the right MFG and bilateral superior frontal gyrus (SFG). The AD group also had a lower probability of tract (POT) between the right MFG and SFG, which was significantly associated with global cognition. These findings suggest that synapse loss contributes to functional and structural connectivity alterations underlying cognitive impairment in AD.

**Figure 2:**
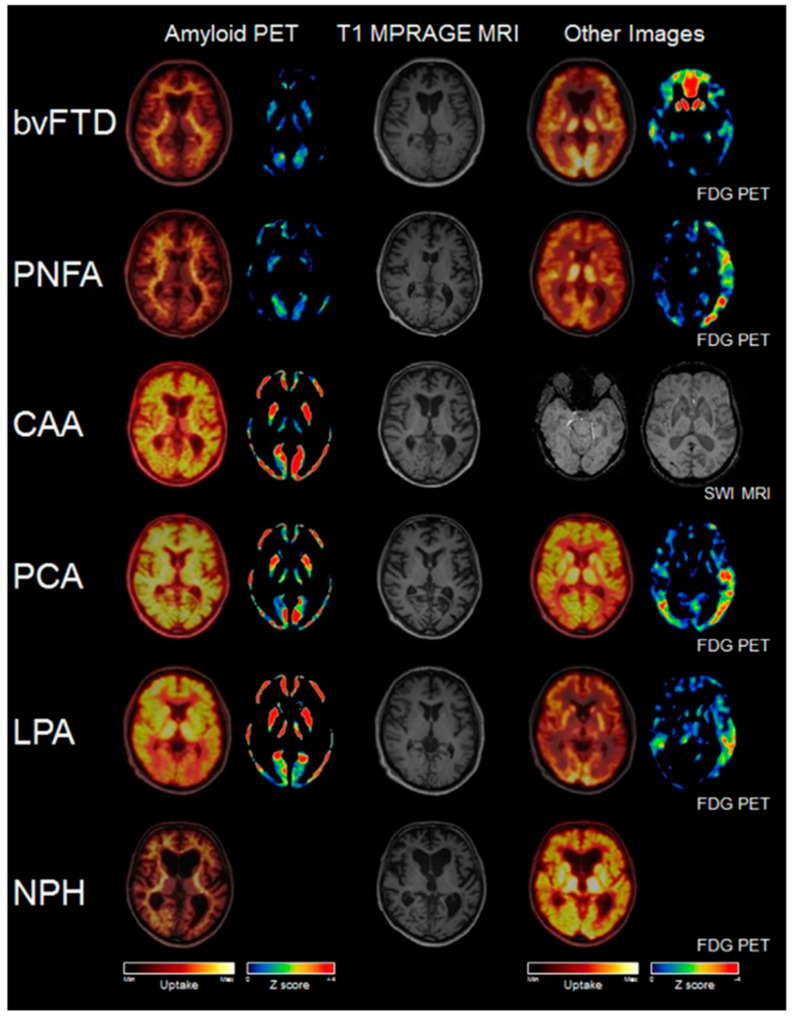
Typical amyloid PET/MRI in rare dementia diseases are shown in the image. The image consists of two columns on the left side, displaying the overlay of 18F-florbetaben amyloid PET/MR. The middle column shows the corresponding anatomical T1-MPRAGE MR slice. The right side of the image displays the corresponding slices of other imaging modalities. The abbreviations used in the image include bvFTD for behavioral-variant frontotemporal dementia, CAA for cerebral amyloid angiopathy, LPA for logopenic aphasia, NPH for normal pressure hydrocephalus, PCA for posterior cortical atrophy, PNFA for progressive nonfluent aphasia, and SWI for susceptibility-weighted MRI [60].

**Figure 3:**
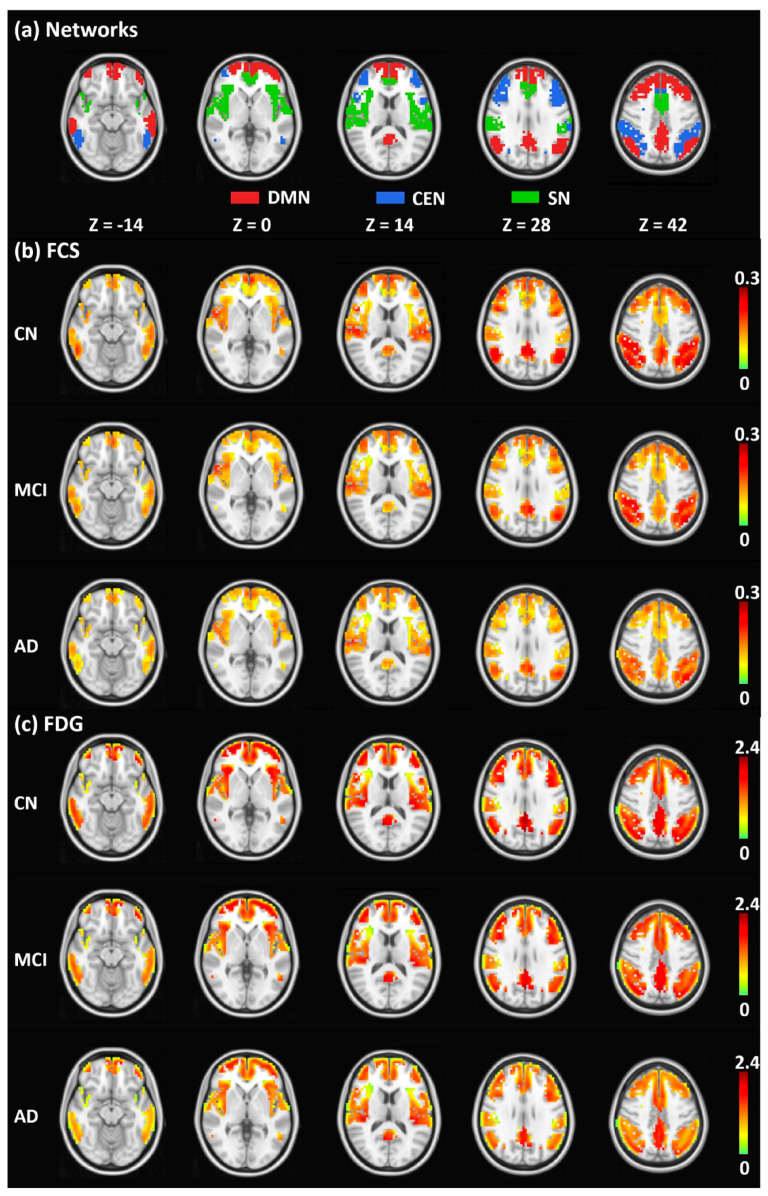
The results of the triple network parcellation and the maps of average functional connectivity strength (FCS) and FDG in the CN, MCI, and AD groups are presented. Using fMRI data, the parcellation results for the DMN (red), CEN (blue), and SN (green) are illustrated in (**a**). The average FCS (**b**) and FDG-SUVR (**c**) maps of the CN, MCI, and AD groups are shown, with the FCS and FDG-SUVR maps masked by the triple-network parcellation results in (**a**). All maps are overlaid on the MNI T1-weighted template. Lower FCS and FDG in MCI and AD can be observed by visual inspection. The abbreviations used in the image include DMN, default-mode network; CEN, central executive network; SN, salience network; FCS, functional connectivity strength; FDG, fluorodeoxyglucose; CN, cognitively normal; MCI, mild cognitive impairment; AD, Alzheimer’s disease [61].

The utilization of PET/MR imaging technology offers several notable advantages in the early diagnosis of AD. Firstly, the synchronous acquisition of PET and MR images enhances the accuracy and efficiency of diagnosis and treatment [58,59]. Secondly, the non-invasive nature of this technology eliminates the need for surgical procedures, thereby minimizing the risk to patients and increasing overall safety. Thirdly, the high-resolution imaging capabilities of PET/MR provide detailed biological biomarkers and anatomical images, facilitating a more precise evaluation of AD (Figure 2 and Figure 3) [60,61]. Lastly, the ability to simultaneously evaluate multiple biological biomarkers enables a comprehensive assessment of the disease. Consequently, the integration of hybrid PET/MR imaging is anticipated to play a pivotal role in improving the early diagnostic accuracy and clinical outcomes for patients with AD.

The different stages of AD include preclinical stage, MCI, mild, moderate, and severe AD. When analyzing PET/MR images, important information that needs to be extracted from the images includes detecting and localizing lesions, measuring volume, assessing lesion heterogeneity, segmenting organs and tissues, quantifying lesions, analyzing functionality and metabolism, and merging PET and MR data. Overall, the analysis of PET/MR images plays a critical role in detecting and characterizing AD, planning treatments, and evaluating organ function and anatomical structures. Compared to traditional methods and machine learning (ML), DL techniques have demonstrated better performance in various medical imaging tasks. DL models can effectively learn from large datasets, automatically extract meaningful features from raw PET/MR images, and this is particularly important for AD analysis due to the availability of significant imaging data. DL models have the potential to capture subtle imaging biomarkers and complex patterns associated with the progression of AD. However, the application of DL methodologies in AD analysis is limited by various challenges and limitations, such as the availability and quality of data, interpretability of results, the risk of overfitting and generalization issues, and the computational requirements.

## 3. Deep Learning Algorithms

Deep learning, an artificial neural network (ANN) technique, replicates the learning mechanism of the human brain through the utilization of multi-layer neural networks. This methodology facilitates the effective processing and analysis of intricate data [62]. Various deep learning algorithms exist (Table 2), encompassing feed forward neural networks (FFNN), convolutional neural networks (CNN), recurrent neural networks (RNN), generative adversarial networks (GAN), autoencoders, and deep reinforcement learning (DRL).

### 3.1. Feed Forward Neural Networks

Feed forward neural network (FFNN) excels in addressing classification and regression problems [63]. Comprising an input layer, hidden layer, and output layer, the FFNN receives raw data in the input layer, extracts feature through the hidden layer, and produces the final prediction outcome in the output layer. Each layer consists of multiple neurons with their own weights and biases, which can be refined through training.

The FFNN’s advantage lies in its ability to handle high-dimensional data and nonlinear relationships, making it suitable for various data types. However, substantial amounts of data and computational resources are required for training, and overfitting is a potential concern. In the medical field, the FFNN has been extensively applied in medical image processing [64], as well as disease prediction and classification, enabling healthcare professionals to provide personalized treatment and preventive measures [65].

### 3.2. Convolutional Neural Networks

The core principle underlying Convolutional Neural Networks (CNN) lies in their ability to extract salient features from input data through convolutional operations [66,67,68,69]. These extracted features are then further refined through pooling operations, resulting in a reduction in the feature map size. Finally, these refined features are mapped onto the output layer through fully connected layers, enabling robust classification and recognition. The widespread adoption and acclaim of CNNs across various domains attest to their remarkable efficacy [70,71].

CNNs have emerged as an indispensable deep learning algorithm, distinguished by their unparalleled ability to automatically unveil intrinsic features and structural patterns from input data. This unique capability empowers CNNs to excel in classification and recognition tasks with exceptional precision [72,73].

### 3.3. Recurrent Neural Networks

Recurrent neural networks (RNN) have gained widespread recognition for their exceptional prowess in processing sequential data. Their defining characteristic lies in the incorporation of recurrent connections, which facilitate the seamless transfer of information across different time steps, thus enabling the modeling and prediction of sequential data [74].

The underlying principle that governs RNNs revolves around the utilization of recurrent neurons to establish intricate relationships among sequential data, thereby capturing the essence of the data through the exchange of information across various temporal instances. In essence, RNNs represent a neural network model that possesses the capability to predict sequential data, and they have found extensive utilization within the domain of medical imaging. With the relentless advancements in artificial intelligence technology, RNNs are poised to assume an increasingly pivotal role in the field of medical imaging in the foreseeable future.

### 3.4. Autoencoder

The autoencoder, a widely used unsupervised learning algorithm, possesses the ability to compress and decompress input data while preserving its essential characteristics [75]. It consists of two essential components: an encoder and a decoder.

The autoencoder holds significant importance in the field of medical imaging, where data often exhibit high dimensionality and complexity, as seen in PET and MRI images. By compressing high-dimensional data into a lower-dimensional representation, it simultaneously reduces computational and storage costs, while improving image quality by reducing the impact of noise and artifacts [76]. The autoencoder serves as a valuable unsupervised learning algorithm that facilitates feature extraction, dimensionality reduction, and image reconstruction [77].

### 3.5. Generative Adversarial Network

Generative Adversarial Network (GAN) is a deep learning algorithm that produces realistic images and data [78]. It consists of a generator and a discriminator. The generator creates realistic outputs using random noise as input, while the discriminator determines the authenticity of the input.

During training, the generator and discriminator compete to improve their abilities. GAN has advantages such as generating high-quality outputs without explicit rules and stronger generation ability than other models [79,80]. It can be used for tasks such as image transformation. However, GAN has drawbacks. Its training process is complex and requires adjusting multiple hyperparameters. It can also suffer from mode collapse, producing limited types of samples. Controlling the generated results can be challenging. In conclusion, GAN is a powerful generative model that produces high-quality outputs. Despite its limitations, advancements in deep learning technology are expected to enhance GAN’s capabilities.

### 3.6. Deep Reinforcement Learning

Deep reinforcement learning (DRL) is a combination of deep learning and reinforcement learning that allows machines to complete tasks without human intervention [81]. It guides behavior through rewards and punishments, selecting actions based on the current state and adjusting strategies to maximize future rewards. DRL uses deep learning models to learn optimal strategies and behaviors.

DRL has advantages in autonomous learning and adaptation, improving performance through trial and error. It has applications in the medical field, autonomous driving, robotics, and gaming. However, DRL requires significant time and computing resources, and faces challenges such as data and resource requirements, uncertain results, and problems such as overfitting and sample bias. In conclusion, DRL is a promising algorithm for autonomous task completion, improving efficiency and safety. As technology advances, DRL will be applied to more fields, bringing convenience and innovation.

## 4. Application of DL in AD PET/MR Imaging

The challenge of early diagnosis and prediction of AD is still significant. Despite using clinical symptoms and imaging examinations, the current methods for prediction lack accuracy. Additionally, the rate and pattern of AD development can differ greatly among individuals, making prediction even more difficult. However, the use of deep learning technology in AD PET/MR imaging is helping to automate early diagnosis and prediction of disease progression trends, as well as enhance medical image analysis and processing. Several studies have utilized DL in PET and MR evaluations of AD (refer to Table 3). The following sections provide a summary of the application of DL in image analysis and diagnosis [82].

### 4.1. Image Segmentation

Image segmentation is the process of dividing an image into smaller sub-regions. In the context of PET/MR imaging, precise segmentation of the human brain is of utmost importance for accurate diagnosis, especially when dealing with AD patient data. Conventional image segmentation methods require manual selection of features and parameters, which can be time-consuming and require significant expertise. However, deep learning has emerged as a highly adaptable approach that can automatically learn features and parameters, leading to more precise classification, segmentation, and prediction tasks for brain imaging data [83,84,85]. Overall, DL provides a promising avenue for automated brain image processing in neuroscience research.

### 4.2. Image Reconstruction

DL is widely used in medical imaging for segmentation, particularly in PET/MR image reconstruction. It improves accuracy and speed, providing precise and rapid support for medical imaging diagnosis.

Deep learning automatically discovers patterns and features in images by learning from a large amount of data, improving the accuracy of PET/MR image reconstruction. Traditional methods are time-consuming and susceptible to noise and interference. By identifying features of different tissues and organs, deep learning can reconstruct images more accurately, enabling early diagnosis of AD. Deep learning optimizes the model structure and algorithm parameters to reduce computational complexity and time consumption, enhancing the speed of PET/MR image reconstruction [16,86,87,88]. This improves efficiency and reduces examination time for patients who cannot cooperate.

### 4.3. Diagnosis and Prediction

The identification of subtle changes in the brain that differentiate AD from normal aging or other neurological conditions is a challenging task in PET and MRI imaging. Traditional methods of disease diagnosis in PET/MR imaging require manual selection of features and parameters, but deep learning can automatically learn features and parameters, uncovering complex latent patterns in MRI and PET. Studies have demonstrated the efficacy of this approach [82,89,90]. Similarly, Zhou et al.’s [91] study demonstrated the effectiveness of amyloid PET/MRI using deep learning techniques (AUC = 0.87 in separating between AD and NC groups; AUC = 0.79 in separating MCI and NC groups; AUC = 0.71 separating AD and MCI groups). This approach also showed better early diagnosis and prediction of AD, providing valuable guidance for clinical practice.

### 4.4. Visualization of Pathological Features

Early diagnosis of AD is crucial for effective treatment to slow down further deterioration. Visualizing the morphological features of early-stage AD is of great clinical value, including the presence of neurofibrillary tangles and amyloid plaques. However, traditional imaging diagnosis methods often require specialized skills and experience, making large-scale data analysis inefficient. A recent study has proposed a novel approach called the Multi-Directional Perceptual GAN (MP-GAN) for visualizing the severity of AD in different stages of patients [92]. By introducing a new multi-directional mapping mechanism into the model, the MP-GAN can efficiently capture significant global features. Compared to traditional manual feature extraction methods, deep learning has the advantages of efficiency, accuracy, and automation in capturing pathological features, improving the accuracy and efficiency of diagnosis. This approach can also be applied to morphological feature analysis of other diseases.

**Table 3:** Summary of the findings of deep learning in the PET and MRI of Alzheimer’s Disease.

Sr. No	Author	Network	Samples	Features	Dataset	Optimal Result	Clinical Implication	Reference
1	Jo et al., 2020	CNN	300	tau PET	ADNI	Accuracy = 90.8%	Potentially aiding in the early detection of AD during its prodromal stages.	[93]
2	Hamghalam et al., 2020	GAN	-	MRI	BraTS’18	Enhances DSCs by approximately 1%	Accurately segments brain tissue, the source code for synthesizing high tissue contrast images is publicly available.	[88]
3	Kim et al., 2021	CNN	1433	FDG and amyloid PET, MRI	ADNI, KBASE	Accuracy = 75.0%, AUC = 0.86	Potential to accurately identify amyloid PET positivity in a clinical setting.	[82]
4	Peng et al., 2021	CNN, GAN	25	Amyloid PET	-	100% classification accuracy	PET imaging workflow can be enhanced by utilizing deep learning-based techniques.	[86]
5	W. Zhang et al., 2021	CNN	2386	FDG PET, MRI and neuropsychological tests	ADNI	Accuracy = 95.6%	Valid diagnoses explained uncertain cases based on neurodegeneration and depression.	[89]
6	Zhou et al., 2021	CNN	355	FDG PET	ADNI	Accuracy = 90.6%	Promising approach for diagnosis of conversion from MCI to AD.	[94]
7	Zou et al., 2021	CNN	766	tau PET	ADNI	Accuracy > 80%	Improve tau PET’s role in early disease and extend the utility of tau PET across generations of radioligands.	[95]
8	Etminani et al., 2022	CNN	757	FDG PET	ADNI and EDLB	AUC = 0.96	DL model predicted common neurodegenerative disorders with performance comparable to human readers and consensus.	[96]
9	Thakur and Snekhalatha, 2022	CNN	1130	FDG PET	ADNI	Accuracy = 98.4%, AUC = 0.95	Help classifying MCI subtypes (EMCI, LMCI) and AD/CN groups from PET brain images.	[90]
10	Q. Zhang et al., 2021	DRL	1349	MRI	ADNI, AIBL and NACC	AUC = 0.99	The model serves as a link between clinical practice and AI diagnosis, offering insight into the interpretability of AI technology.	[97]
11	Hui et al., 2023	DRL	-	-	-	-	DRL holds great potential in the detection and prediction of AD progression.	[98]
12	Marti-Juan et al., 2023	autoencoder	897	PET and MRI	ADNI, synthetic data	Reducing error by 5%.	Produce authentic synthetic trajectories of imaging biomarkers from cognitive assessments.	[87]
13	Choi et al., 2020	CNN	636	FDG PET	ADNI	AUC = 0.94	Distinguish individuals with PD who also had dementia.	[99]
14	Cui et al., 2019	RNN, CNN	830	MRI	ADNI	Accuracy = 91.3%	Great potential in analyzing longitudinal MR images.	[100]
15	Rajasekhar, 2023	FFNN	-	MRI	ADNI	Accuracy = 98.4%	Great importance for early-stage AD prediction.	[17]
16	Yu et al., 2022	GAN	5316	MRI	ADNI	-	The performance of GAN to visualize the subtle lesions in AD diagnosis.	[92]
17	Zhang et al., 2021	CNN	2386	PET and MRI	ADNI	Accuracy = 95.65%	It exhibited clinical validity and possessed the potential for application.	[89]

Abbreviations: CNN convolutional neural networks, GAN generative adversarial network, DRL deep reinforcement learning, RNN recurrent neural networks, FFNN feed forward neural networks, PET positron emission tomography, MRI magnetic resonance imaging, ADNI Alzheimer’s Disease neuroimaging initiative, BraTS’18 multimodal brain tumor segmentation challenge 2018, KBASE Korean Brain Aging Study for the Early diagnosis and prediction of Alzheimer’s disease, EDLB the European Dementia with Lewy Bodies (EDLB) Consortium, NACC national Alzheimer’s coordinating center, AIBL Australian imaging biomarker and lifestyle flagship study of aging, DSC the Dice Similarity Coefficients, AUC area under the curve, AD Alzheimer’s Disease, MCI mild cognitive impairment.

**Figure 4:**
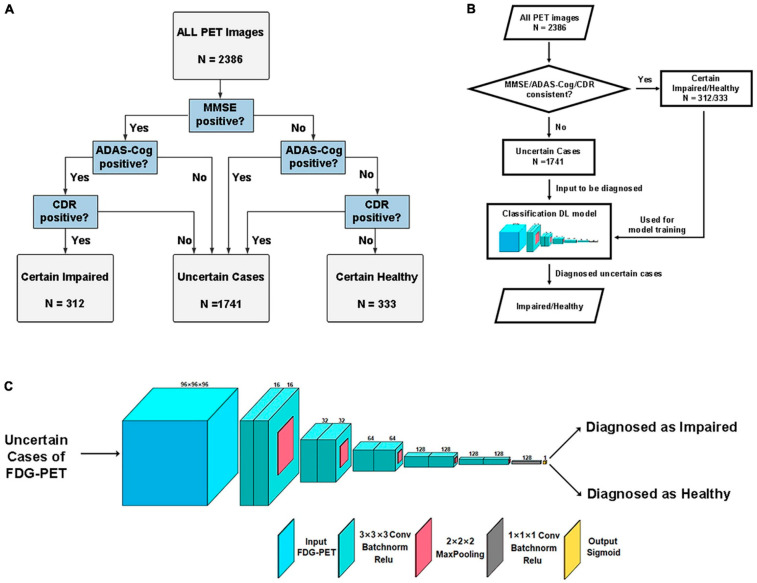
(**A**) A flowchart illustrating the criteria for grouping data based on memory scores from these tests. (**B**) A flowchart depicting the different stages of this research, which include grouping, training, and utilizing a DL model. The final step involves using this framework to diagnose uncertain samples. (**C**) The specific architecture of the 3D CNN model that was designed. The Mini-Mental State Examination (MMSE), Alzheimer’s Disease Assessment Scale-Cognitive Subscale (ADAS-Cog), and Clinical Dementia Rating (CDR) are utilized in this process [89].

Following is a case of DL technologies in real clinical practice [89]. Neuropsychological testing is an important basis for the diagnosis of memory impairment in AD. However, multiple memory tests may generate conflicting results within the subject and lead to uncertain diagnoses in certain cases. This study proposes a DL framework for diagnosing uncertain cases of memory impairment of AD. A total of 2386 samples from the Alzheimer’s Disease Neuroimaging Initiative (ADNI), which included individuals with Alzheimer’s disease (AD), mild cognitive impairment (MCI), and cognitive normal (CN) were recruited. All raw data of PET and MRI images were obtained using the standardized ADNI protocols and underwent the same processing criteria, PET images were registered to the corresponding MRI. Three different neuropsychological tests, namely the Mini-Mental State Examination (MMSE), Alzheimer’s Disease Assessment Scale-Cognitive Subscale (ADAS-Cog), and Clinical Dementia Rating (CDR) (Figure 4A).

The trained DL framework was utilized to classify all images in the uncertain group as either memory-impaired or healthy. The entire process is illustrated in Figure 4B. A CNN model specifically designed for this purpose was utilized to categorize impaired or healthy individuals based on the images of each case. The structure of the model can be seen in Figure 4C. To introduce non-linearity, a 3D convolution layer with a size of 3 × 3 × 3 and stride 1 was employed, followed by a batch normalization layer and a rectified linear unit (ReLU) activation layer. The downsampling was achieved using 3D max-pooling with a size of 2 × 2 × 2. The number of filters increased progressively from 16 to 128 during the downsampling process. Finally, a 1 × 1 × 1 3D convolution was executed to summarize the high-dimensional features, concluding with a dense layer that utilized sigmoid activation for classification output. For certain cases in the testing set, the proposed DL framework outperformed other methods with 95.65% accuracy. Demonstrated through the longitudinal tracking of its diagnoses, it exhibited clinical validity and possessed the potential for application.

## 5. Future Directions

With the extension of human lifespan and the increasing trend of social aging, AD has become a pressing public health concern. Early diagnosis and intervention have been proven to effectively slow down the progression and symptoms of AD, highlighting the importance of imaging diagnosis and assessment in clinical medicine. The following is a summary of the future direction of the application of deep learning in PET/MR for AD.

### 5.1. Automated Diagnosis

AD diagnosis currently relies on subjective clinical experience, leading to a high risk of misdiagnosis. Automated analysis of PET/MR image data using deep learning technology can provide higher accuracy and faster speed, reducing the workload of doctors. It has broad application prospects and will become an essential tool in medical image diagnosis, supporting early diagnosis and treatment of neurodegenerative diseases.

### 5.2. Predictions of Models

Multi-modal images may be incomplete, leading to a reduction in sample size. Deep learning frameworks such as TPA-GAN and PT-DCN can interpolate and classify multi-modal brain images. Reversible GAN models can reconstruct missing data, and 3D CNN classification models with multi-modal inputs can aid in AD diagnosis. These methods perform well in terms of prediction accuracy and biological interpretability.

### 5.3. Personalized Medicine

Personalized medicine seeks to offer specialized medical services by taking into account a patient’s distinct genetics, environment, and lifestyle. In the treatment of Alzheimer’s disease, deep learning technology can examine and diagnose PET/MR imaging data, leading to enhanced accuracy in evaluating the patient’s condition and predicting disease advancement. Moreover, it can aid in creating customized treatment strategies by considering variations in patients’ genetic information, lifestyle choices, and exercise routines. Ultimately, personalized medicine contributes to the early detection and diagnosis of diseases.

## 6. Conclusions

In conclusion, the application of PET/MR imaging and deep learning algorithms has shown great potential in the early diagnosis and prediction of AD. Deep learning algorithms, such as FFNN, CNN, RNN, autoencoder, GAN, and DRL, have greatly improved the accuracy and speed of image reconstruction, efficient image segmentation, diagnosis, and visualization of AD pathological features.

In the future, the integration of multimodal imaging and automated diagnosis of medical images can further enhance the accuracy and efficiency of AD diagnosis. Predictive models based on PET/MR imaging data can also provide valuable insights into disease progression and response to treatment. Personalized medicine can also be achieved through the development of individualized treatment plans based on patient-specific imaging data.

Overall, the application of deep learning in PET/MR imaging has revolutionized the field of AD diagnosis and treatment. Further research and development are needed to address existing challenges and realize the full potential of these technologies in clinical practice.

## Figures and Tables

**Table 1:** Categorization of PET/MR Imaging in AD (including deep learning approaches).

	Advantages	Drawbacks
Structural MRI	Provides detailed anatomical information. Can detect changes in brain structure, such as atrophy or cortical thinning.	Limited sensitivity in detecting early stages of AD. Difficulty in distinguishing AD-specific changes from normal age-related changes.
Functional MRI (fMRI)	Measures brain activity and connectivity. Can identify functional abnormalities in AD, such as changes in resting-state networks.	Limited specificity in distinguishing AD from other neurodegenerative disorders. Relatively low spatial resolution compared to other imaging modalities.
Positron Emission Tomography (PET)	Can detect specific biomarkers associated with AD, such as beta-amyloid plaques and tau tangles. Provides quantitative measurements of biomarker distribution.	Expensive and time-consuming procedure. Requires the use of radiotracers, which may have limited availability. Ionizing radiation exposure.
PET/MRI Fusion	Combines the strengths of both PET and MRI modalities. Provides complementary information on both functional and structural aspects.	Limited availability and high cost of PET/MRI scanners. Increased complexity in data acquisition and processing.
Deep Learning (DL)	Can extract complex patterns and features from large imaging datasets. Enables automated analysis and classification of AD-related imaging biomarkers. Potential for improving diagnostic accuracy and early detection.	Requires large amounts of labeled training data. Vulnerable to overfitting if the dataset is not representative. Lack of interpretability, making it challenging to understand the underlying biological mechanisms.

**Table 2:** Typical Deep learning algorithms approaches in AD.

	Advantages	Drawbacks
Feedforward Neural Networks (FFNN)	FFNN is a simple and straightforward approach for AD disease classification. Can handle high-dimensional data and has good generalization capability.	FFNN may struggle with capturing temporal dependencies in AD progression. It may also be prone to overfitting if the dataset is small.
Convolutional Neural Networks (CNN)	CNNs are effective in extracting spatial features from images or volumetric data. Can automatically learn relevant features and hierarchies, making them well-suited for image-based AD analysis.	CNNs may not effectively capture temporal information, which is crucial for understanding AD progression over time. May also require a large amount of labeled training data.
Recurrent Neural Networks (RNN)	RNNs are designed to handle sequential data and can capture temporal dependencies effectively. Can model the dynamics of AD progression over time and handle variable-length input sequences.	RNNs may suffer from the vanishing gradient problem, making it difficult to capture long-term dependencies. Can also be computationally expensive and require significant resources for training.
Autoencoder	Combines the strengths of both PET and MRI modalities. Provides complementary information on both functional and structural aspects.	Autoencoders are primarily unsupervised learning models and may not directly handle AD classification tasks. May also struggle with capturing complex relationships between features.
Generative Adversarial Networks (GAN)	GANs can generate synthetic data samples that resemble real AD data. Can be used for data augmentation, increasing the size of the training dataset. GANs can also be used for anomaly detection in AD diagnosis.	GANs can be challenging to train and may suffer from mode collapse or instability. May also generate unrealistic samples that do not accurately represent the AD disease characteristics.
Deep Reinforcement Learning (DRL)	DRL can be used to optimize treatment strategies for AD patients by learning from trial and error. Can adapt and improve treatment decisions based on patient feedback, leading to personalized and adaptive therapies.	DRL requires a substantial amount of training data and can be computationally expensive. May also be challenging to define a suitable reward function for AD treatment, and the learned policies may not generalize well to new patients.

Note: The categorization and pros/cons provided above are general observations and may vary depending on specific implementations and variations in the mentioned DL approaches in AD research.

## Data Availability

No new data were generated or analyzed in support of this research.
